# Determinants of exclusive breastfeeding practice to infants aged less than six months in Offa district, Southern Ethiopia: a cross-sectional study

**DOI:** 10.1186/s13006-016-0091-8

**Published:** 2016-12-09

**Authors:** Ayele Lenja, Tsegaye Demissie, Bereket Yohannes, Mulugeta Yohannis

**Affiliations:** 1 Integrated Family Health Program, Wolaita Sodo Cluster Office, Wolaita Sodo, Ethiopia; 2 Public Health Nutrition, Wolaita Sodo University, Wolaita Sodo, Ethiopia; 3 School of Public Health, Wolaita Sodo University, Wolaita Sodo, Ethiopia; 4 University of Bergen, Bergen, Norway; 5 Hawasa University, Awassa, Ethiopia

**Keywords:** Determinants, Exclusive breastfeeding, Southern Ethiopia

## Abstract

**Background:**

World Health Organization (WHO) recommends timely initiation of breastfeeding after birth and only feeding breast milk to infants during the first 6 months of life. It was estimated that exclusive breastfeeding can reduce infant deaths by 13%. The practice of exclusive breastfeeding is suboptimal in many parts of Ethiopia to a varied extent. Factors associated with exclusive breastfeeding practice and the proportion of its practice was not well documented in Offa district. Therefore, this study was aimed to assess the determinants of exclusive breastfeeding in the first 6 months of life in Offa district, Southern Ethiopia.

**Methods:**

A community-based cross-sectional study was carried out in 396 mothers of infants younger than 6 months using random sampling. Data were collected from mothers of the infants by trained interviewers. Exclusive breastfeeding was measured by the history of infant feeding in the prior 24 h. Pretested and structured questionnaires adopted from standard questionnaires and Ethiopia linkages modules were used. Multivariate logistic regression was used to identify factors significantly influencing exclusive breastfeeding practice.

**Results:**

Based on findings of this study of 396 participants, the proportion of exclusive breastfeeding was 78.0% and awareness of exclusive breastfeeding and bottle feeding were 85.6 and 6.1% respectively. About 6% of infants were given prelacteal feeds. The number of infants fed cow milk was 12.9%, formula 7.8%, water 8.5%, fruits and semisolids 1.5%, over 24 h prior to the survey. The initiation of breastfeeding within one h (AOR 2.2; 95% CI 1.1, 4.27), attending formal education (AOR 4; 95% CI 2.20, 7.25), having an awareness on the benefits of exclusive breastfeeding (AOR 6; 95% CI 3.10, 11.70) and knowledge of colostrum feeding (AOR 2.1; 95% CI 1.11, 4.27) had a statistically significant association with exclusive breastfeeding in the study area.

**Conclusions:**

The practice of exclusive breastfeeding as well as awareness was worthy in Offa district. Additionally the proportion of bottle feeding use was small. However, feeding other than breast milk was associated with the perception that breast milk alone was insufficient for their child. Strategies on promoting exclusive breastfeeding practice must focus on strengthening women’s education and awareness creation activities further.

## Background

Globally infant and young child deaths occur mainly due to inappropriate infant feeding practices and infectious diseases. Directly or indirectly, malnutrition has been responsible for 60% of 10.9 million under five deaths [1]. More than two third of these deaths were often associated with inappropriate feeding practices during the first year of life. In order to reduce infant and young child mortality, exclusive breastfeeding has been recognized as one of the major interventions worldwide [2–5].

Breastfeeding has many health benefits for both mother and infant [6–8]. Breast milk contains all the nutrients an infant needs in the first 6 months of life. It is the most ideal, safe, and complete food that a mother can provide for her newborn. Therefore, WHO recommends infants should be exclusively breastfed for the first 6 months of life to achieve optimal growth, development and health [1]. Moreover, the early initiation of breastfeeding and feeding of colostrum provides a natural immunity against many infections [9]. Hence, initiation of breastfeeding in the first 1 h of life is also recommended as it reduces infant mortality significantly [10].

Breastfeeding is almost universal practice in Ethiopia. However, only 52% of children aged <6 months were exclusively breastfed. In addition to breast milk 19, 14, 4 and 16% of infants <6 months were given plain water only, milk other than breast milk, non-milk liquids and fed using a bottle respectively [11]. Nationally nearly 52% of infants initiated breastfeeding within one h of birth and this varies in different settings [11, 12]. The initiation of breastfeeding within one h was lowest in Amhara region (38%) and highest in the Southern region (67%) based on latest Ethiopian Demographic and Health Survey (EDHS) report [11].

Multiple factors can influence the patterns of exclusive breastfeeding and early initiation of breastfeeding to infants. Based on the reports of a study done at Halaba special district; antenatal care, postnatal care, initiation of breastfeeding immediately within 1 h, having attended formal education and awareness about exclusive breastfeeding had a statistically significant influence with the practice of exclusive breastfeeding [13]. Appropriate exclusive breastfeeding in the first 6 months of life lowers child mortality extensively based on a clinical trial done in developing countries [14]. The latest EDHS shows breastfeeding within one h of birth does not vary significantly by the type of assistance at delivery. The likelihood that a child is breastfed in the first h after birth increases with the mother’s educational status and wealth quintile [11]. The other finding indicate significant correlation between exclusive breastfeeding and length for age Z-score [15].

To encourage an improvement of child feeding practices, additional research is needed to explore the gaps for an intervention. Such evidence is limited in southern Ethiopia on the predictors of exclusive breastfeeding practice. Moreover, in Offa district, other than reports of organizations working in that specific district, valuable findings were not recorded. Therefore, this study was intended to explore the level of exclusive breastfeeding practice and to identify predictors of exclusive breastfeeding in Offa district, Southern Ethiopia. The findings will assist the promotion of appropriate infant and young child feeding practices at the study setting and other similar settings.

## Methods

### Study area

This study was conducted in Offa district, is located at 159 Km from regional capital Hawasa and 380 km south from Addis Ababa. Offa is one of 12 rural districts in Wolaita province (Zone) of Southern Ethiopia. The district has 23 kebeles (the lowest administrative units next to district). Based on projection from the 2007 population and housing census report, the total population in 2014/15 is estimated to be 131,708. Most of the population resides in rural setting of this district 124,306 (94.3%). The estimated number of infants aged <6 months was 2076 (1.7%) in 2013/14. The health facility distribution comprises of 4 health centers, 25 health posts, 7 private clinics, and 4 pharmacy vendors based on reports of zonal health department.

### Study design

A community based cross-sectional study was employed from February to April in 2015 at rural communities of Offa district, in Southern Ethiopia.

### Source and study population

All mothers with infants <6 months residing in Offa district were the sourced population and mothers with infants <6 months included in the study were study population.

### Inclusion criteria and exclusion criteria

All mothers of infants <6 months old were included and mothers who were seriously ill and/or had difficulty communicating were excluded from the study.

### Sample size and sampling procedure

The sample size was determined by using assumptions for single population proportion formula considering the anticipated proportion of exclusive breastfeeding of infants <6 months as 52% from EDHS 2011 [11]. The level of confidence was fixed as 95% and with 5% margin of error. An non-response rate of 5% was taken for this study. Therefore, the total calculated sample size for this study was 403 mothers with infants <6 months. Ten kebeles were randomly selected from the list of 23 kebeles. The sample is then distributed in randomly selected kebeles by population proportion to size. Thus, using a registered list of family with infant <6 months at the health posts, individual mothers having an infant <6 month of age were selected using systematic random sampling technique.

### Data collection

Data were collected by face-to-face interview of the selected mother by home visits, using the pretested and structured questionnaire. Pretesting was done on 5% of the total respondents at none of the selected kebeles and corrections were made. If the eligible mother was absent from the home at the time of data collection, a revisit was done and a mother who was absent at second visit were considered as non-respondent. Age of the child was calculated from the date of birth to the date of the survey. For those with written evidence, the date of birth was obtained from vaccination cards. When a mother had no written documents, their child’s age was established on the date given by the mother triangulated with local calendars.

Data collectors and supervisors were trained on data collection tool and its procedures for 3 days. Data were collected by five Bachelor of Science (BSc) degree graduates, experienced in enumeration activities and fluent in the local language. Data quality was assured through pretesting and cross-checking the questionnaires daily by supervisors for completeness and consistency. Problems identified during data collection were discussed overnight with data collectors and supervisors.

### Operational definitions

#### Early initiation of breastfeeding

The proportion of children born in the last 6 months and breastfed within 1 h of birth.

#### Exclusive breastfeeding

Breastfeeding while giving no other food or liquid, not even water, with the exception of drops consisting vitamins and mineral supplements for 24 h prior to this survey.

#### Prelacteal feeds

Include any food, liquid, or herbs given before initiation of breastfeeding.

#### Bottle feeding

Feeding of any liquid/semisolid including breast milk from a bottle with a teat.

### Data analysis

After proper collection, data were entered in to Epi Info 3.5.1 statistical package. Then it was exported to SPSS version 16 for cleaning and analysis. Descriptive statistics were carried out for all variables including the level of exclusive breastfeeding in infant <6 months. Chi-square test of proportion and binary logistic regressions were used to measure the strength of association among each independent and dependent variable. Variables with *p*-value <0.25 in the bivariate analysis were considered as candidates for the final model. Multivariate logistic regressions were conducted to identify the predictors of exclusive breastfeeding practice and *p*-value <0.05 was considered as the cutoff point for statistical significance.

## Results

### Sociodemographic characteristics

A total of 396 mothers with infants <6 months of age participated in the study with a response rate of 98%. The age range of mothers included in the study was 15–40 years. Of the total participants 32 (8.4%) were young mothers aged 15–19 years and 12 (3.2%) were aged 35 and above. The median age of mothers was nearly 26 years and age ranges of the children considered in this study were 0–5 months (optimal age range for exclusive breastfeeding). The distribution of children in age group was; 118 (29.8%), 78 (19.7%), 75 (18.9%), and 125 (31.6%) for <2 months, ≥ 2 up to <3 months, ≥ 3 months up to <4 months and ≥4 up to 5 months respectively. The median age of the children was 4 months and 205 (51.8%) children were male from the total study participants. Almost half (47%) of the respondent mothers had not attended formal education, 357 (90.2%) of respondents were housewives, 20 (5.1%) were private business women, 11 (2.8%) were staff in government and non-government offices and 2 (0.5%) were farmers (Table 1).

**Table 1:** Socio-demographic characteristics of respondents in Offa district, 2015

Variables	Frequency (*n*)	Percent (%)
Age of mothers in years		
15–24	118	29.8
25–34	250	63.1
≥35	28	7.1
Age of children in months		
≤1 months	118	29.8
2 months	78	19.7
3 months	75	18.9
4–5 months	125	31.6
Mother attended formal education		
Yes	210	53.0
No	186	47.0
Husband attended formal education		
Yes	284	71.9
No	111	28.1
Average monthly income		
0–500 Birr	187	47.2
501–1000Birr	17	4.3
1001 and above	17	4.3
Don’t know	175	44.2
Household member (family size)		
1–5 members	214	54.0
>5 members	182	46.0
Mothers occupation		
Housewife	357	90.2
Private business	20	5.1
Farmer	2	0.5

### Health service and breastfeeding related characteristics

The present study indicates 266 (67.2%) and 221 (55.8%) mothers had followed antenatal and postnatal care respectively. The mean (SD) number of times mothers attended antenatal care was 3.15 (0.95) throughout the gestational age and 140 (35.4%) mothers gave birth to the index children in health facilities, the rest, 256 (64.6%) delivered at home (Table 2).

**Table 2:** Distribution of characteristics related to breastfeeding in mothers at Offa district, 2015

Variables	Frequency (*n*)	Percent (%)
Place of delivery		
Home	256	64.6
Health Institution	140	35.4
Preceding birth interval		
First child	22	5.6
Less than 2 years	253	63.9
Two years and above	104	26.3
Don’t know	17	4.3
Birth order of child		
First child	103	26
2nd – 4th	191	48.2
5th and above	102	25.8
Main reason for not EBF (*n* = 87)		
Breast milk alone was insufficient	56	64.4
Mother left home	10	11.5
Age of weaning	6	6.9
Necessary for health	5	5.3
Mother sick	1	1.1
Other	9	10.3
Bottle feeding practice		
Yes	24	6.1
No	372	93.9
Exclusive breastfeeding		
Yes	309	78.0
No	87	22.0
Frequency of breastfeeding		
≥8 times per day	318	80.5
<8 times per day	77	19.5

Among those who ever breastfed, more than half 282 (71.2%) initiated breastfeeding within the first h of delivery and 305 (77.0%) gave colostrum to their infants, and 16 (4%) of mothers provided clean water, 7 (1.8%) gave rue (tena-adam) to infants with the perception of protecting children against illness. About 318 (80.5%) mothers breastfed their infants ≥8 times per day while 77 (19.5%) of the mothers breastfed their children < 8 times a day. Moreover, 339 (85.6%) mothers have knowledge about exclusive breastfeeding practices (Table 2).

### 24-hour recall of selected child ate over 24 hours

Prior to interview 51 (12.9%), 33 (8.5%), 31 (7.8%) and 6 (1.5%) infants were fed cow milk, water, formula feed and semisolids respectively within 24 h. Moreover, 393 (99.2%) infants were fed breast milk over the same period. But, about 309 (78.0%) mothers reported to have exclusively breastfed (only breast milk) their children for the last 24 h. The main reason that mothers fed their infants other than breast milk was the perception that breast milk alone was insufficient and perception that it is necessary for their infant’s health and growth.

### Practice of exclusive breastfeeding related with some sociodemographic characteristics

Among mothers of children aged ≤1 month, 112 (94.9%) exclusively breastfed their infants, among mothers of children aged 2–3 months, 129 (84.3%) exclusively breastfed and among mothers of children aged 4–5 months, 68 (54.4%) exclusively breastfed their children. This shows the proportion of exclusive breastfeeding declines as children gets older.

### Factors associated with exclusive breastfeeding at Offa district

To identify factor associated with exclusive breastfeeding in children aged <6 months, initially bivariate analysis was done to select candidiate variables for multivariate regression. The result shows; that maternal education, husbands education, antenatal care, postnatal care, colostrum feeding, early initiation of breastfeeding within one h after delivery, and mothers who had received information on benefits of exclusive breastfeeding were associated with the practice of exclusive breastfeeding, compared to their respective counterparts. Similarly there was no significant disparity in exclusive breastfeeding practice among mothers who have male or female children.

Multivariate logistic regression was used to identify predictors after controlling confounders. All variables that have *p*-value <0.25 at bivariate analysis were entered in to final model. The output of the final model shows; maternal education, feeding colostrum, early initiation of breastfeeding and having knowledge on exclusive breastfeeding remained strong influencers of EBF practice. Mothers who can read and write (AOR 4; 95% CI 2.20, 7.25), fed colostrum (AOR 2.1; 95% CI 1.11, 4.28), initiated breastfeeding immediately after birth (AOR 2.2; 95% CI 1.1, 4.27) and have knowledge on exclusive breastfeeding (AOR 6; 95% CI 3.1, 11.7) were more likely to exclusively breastfeed their children than their respective counterparts (Table 3).

**Table 3:** Multivariate analysis of variables predicting exclusive breastfeeding practice

Variables	Practiced EBF		AOR (95% CI)	*p*_value
	Yes	No		
Age of the mother				
15–24	96	22	0.65 (0.21,1.99)	0.45
25–34	192	58	0.63 (0.23,1.74)	0.37
≥34	21	7	1	
Mothers read and write				
Yes	189	21	4.0 (2.20,7.25)	0.001**
No	120	66	1	
Husband education				
Yes	236	48	0.90 (0.48,1.89)	0.90
No	72	39	1	
ANC attendance (ANC)				
Yes	223	43	0.88 (0.45,1.69)	0.69
No	44	88	1	
Postnatal care (PNC)				
Yes	188	33	0.81 (0.42,1.53)	0.53
No	121	54	1	
Initiation of breastfeeding				
Within 1 h	238	44	2.2 (1.1,4.27)	0.001**
After 1 h	71	43	1	
Colostrum fed				
Yes	256	49	2.1 (1.11,4.28)	0.023*
No	53	33	1	
Awareness of EBF				
Yes	288	51	6.0 (3.1,11.7)	0.001**
No	21	36	1	

^*^ *p*-value <0.05 (significantly associated) ^**^ *p*-value <0.001 (highly significant association)

## Discussion

The result shows 309 (78.0%) mothers breastfed their children exclusively which is similar to studies done in Oromia region (71.3%) and another similar study done in afar reported 81.1% of exclusive breastfeeding practice in under 6 month age children [16]. However the finding is higher than the national percentage of 52% exclusive breastfeeding in Ethiopia and also a study from Debremarkos [11, 17]. The reason for a relative large percentage might be the progress with implementing WHO recommendations in the study area.

As indicated elsewhere, 68 (54%) of infants among 4–5 months were breastfed exclusively. This finding is higher than findings of a study done in Goba district (17%), the national months’ specific exclusive breastfeeding for age group 4–6 months (32%), and the findings of a survey conducted on the practice of infant feeding and the influencing factors in United Arab Emirates (46%) [18–20]. The level of exclusive breastfeeding declines as infant get older. This appears to be due to the fact that in the rural community, a special postpartum care is usual in the first few months after birth where mothers remain at home, creating a good opportunity to exclusively breastfeed their infant. Then mothers become busy with their day to day activities to assist their household as the age of child increases. Additionally, mothers also perceive breast milk is insufficient as child gets older.

Mothers who attended formal education were four times more likely to exclusively breastfeed their children than their counterparts. The finding supports the opinion in a parallel study at Nassarawa state, that maternal education is as important as knowledge about nutrition and hygienic practice [21]. A similar study from United Arab Emirates also indicates a positive effect of maternal education on breastfeeding [22]. Moreover the finding also agrees that maternal education is a key predictor for child feeding practices in developing countries [23]. This can be explained by the fact that a mother who attended formal education has also been exposed to information related to exclusive breastfeeding through different kinds of channels like; posters, family health cards and other electronic information that can support the exclusive breastfeeding practice.

Furthermore, mothers who discarded colostrum were more likely not to exclusively breastfeed their children than those who breastfed from birth. The finding was in line with the results of a qualitative study from Ghana [24]. Additionally, the findings of this study showed that mothers who have adequate information on exclusive breastfeeding were six times more likely to exclusively breastfed their children than those who have no information. This agrees with the report from Debremarkos that indicated similar associations [17]. The associated factors draw attention to the fact that an awareness of exclusive breastfeeding determines on practice of breastfeeding.

### Study limitations

Infant feeding practices are typically assessed by mother’s report which may lead to recall bias.

## Conclusion

The proportion of exclusive breastfeeding in the district was below WHO Infant and Young Child Feeding recommendations. The practice of exclusive breastfeeding in mothers of under 6 months children was influenced by awareness of the benefit of exclusive breastfeeding, early initiation of breastfeeding, a mother’s education, and feeding colostrum. Feeding other than breast milk in the 24 h recall was associated with a mother’s lack of awareness of the benefit of exclusive breastfeeding and the perception of breast milk was insufficient for their child.

Stakeholders must work intensively on a community based behavioral change and to further promote the benefit of exclusive breastfeeding practices. Mother’s education was the predictor of exclusive breastfeeding and education as whole and women’s educations in particular should be strengthened. At the community level, health extension workers must continue teaching the key messages of exclusive breastfeeding, when visiting mothers at home and at diverse contact points. Health extension workers should mobilize the community using the health development army as key actors to transform views on breastfeeding practices in the study area towards achieving WHO recommendations in the immediate future.

## Abbreviations

ANC: Antenatal care
AOR: Adjusted odds ratio
EBF: Exclusive breastfeeding
EDHS: Ethiopia Demographic and Health Survey
PNC: Postnatal care
WHO: World Health Organization
